# Pilot randomised controlled trial of a remotely delivered online intervention for adolescent mental health problems in India: lessons learned about low acceptability and feasibility during the COVID-19 pandemic

**DOI:** 10.1192/bjo.2022.624

**Published:** 2022-12-27

**Authors:** Pattie P. Gonsalves, Bhargav Bhat, Rhea Sharma, Abhijeet Jambhale, Bindiya Chodankar, Mamta Verma, Eleanor Hodgson, Helen A. Weiss, Baptiste Leurent, Kate Cavanagh, Christopher G. Fairburn, Pim Cuijpers, Daniel Michelson, Vikram Patel

**Affiliations:** PRIDE, Sangath, India; and School of Psychology, University of Sussex, UK; PRIDE, Sangath, India; Medical Research Council Tropical Epidemiology Group, Faculty of Epidemiology and Population Health, London School of Hygiene & Tropical Medicine, UK; School of Psychology, University of Sussex, UK; Department of Psychiatry, University of Oxford, UK; Department of Clinical Psychology, Vrije Universiteit, The Netherlands; School of Psychology, University of Sussex, UK; and Department of Child and Adolescent Psychiatry, Institute of Psychiatry, Psychology and Neuroscience, King's College London, UK; Department of Clinical Psychology, Vrije Universiteit, The Netherlands; and Department of Global Health and Social Medicine, Harvard Medical School, Massachusetts, USA

**Keywords:** Randomised controlled trial, psychosocial interventions, low- and middle-income countries, information technologies, anxiety disorders

## Abstract

**Background:**

‘POD Adventures’ is a gamified problem-solving intervention delivered via smartphone app, and supported by non-specialist counsellors for a target population of secondary school students in India during the COVID-19 pandemic.

**Aims:**

To evaluate the feasibility and acceptability of undertaking a randomised controlled trial of POD Adventures when delivered online with telephone support from counsellors.

**Method:**

We conducted a parallel, two-arm, individually randomised pilot-controlled trial with 11 secondary schools in Goa, India. Participants received either the POD Adventures intervention delivered over 4 weeks or usual care comprising information about local mental health services and national helplines. Outcomes were assessed at two timepoints: baseline and 6 weeks post-randomisation.

**Results:**

Seventy-nine classroom sensitisation sessions reaching a total of 1575 students were conducted. Ninety-two self-initiated study referrals (5.8%) were received, but only 11 participants enrolled in the study. No intervention arm participants completed the intervention. Outcomes at 6 weeks were not available for intervention arm participants (*n* = 5), and only four control arm participants completed outcomes. No qualitative interviews or participant satisfaction measures were completed because participants could not be reached by the study team.

**Conclusions:**

Despite modifications to address barriers arising from COVID-19 restrictions, online delivery was not feasible in the study context. Low recruitment and missing feasibility and acceptability data make it difficult to draw conclusions about intervention engagement and indicative clinical outcomes. Prior findings showing high uptake, adherence and engagement with POD Adventures when delivered in a school-based context suggest that an online study and delivery posed the biggest barriers to study participation and engagement.

Mental disorders are a leading cause of disability among young people worldwide.^1^ Most mental health problems begin by 14 years of age, and one in five adolescents experiences a mental health disorder each year.^1–3^ Despite growing evidence for effective psychosocial interventions, most young people do not receive appropriate help, particularly in low- and middle-income countries (LMICs), which contain 90% of the world's population aged under 25 years.^4,5^

## Impacts of the COVID-19 pandemic

Public health measures, such as lockdowns and school closures, have severely affected many adolescents during the COVID-19 pandemic, despite relatively little mortality and morbidity arising directly from infection.^6–9^ Schools were partially or fully closed for extended periods in many LMICs, coinciding with disrupted service provision more generally (e.g. to social care and specialist mental health services). There have been considerable psychosocial impacts linked to suspended routines and recreation, and rising concerns for family income and health.^1^ The pandemic has also been linked with rising incidence of some mental disorders among adolescents and exacerbations in pre-existing mental health problems.^10–12^

COVID-19 disruptions have accelerated the transition to online delivery of mental healthcare.^13,14^ Reviews of digital mental health interventions have consistently raised concerns about the accessibility and reach of digital technologies, especially among disadvantaged groups,^15^ and difficulties in keeping participants engaged irrespective of social background.^16^ Promising engagement approaches have recently emerged (e.g. ‘gamified’ interventions that offer game-like feel and relatable, interactive content^16,17^); however, evidence is scarce on effectiveness and uptake, especially in LMICs. A recent review of 18 systematic reviews and meta-analyses of digital mental health interventions for adolescents found no studies from low-resource settings.^18^ Another comprehensive review of 83 studies of digital mental health interventions for children and young people found only one report from an LMIC.^19^

## The current study

The current study describes a pilot feasibility and acceptability trial of ‘POD Adventures’, a gamified problem-solving intervention delivered via a smartphone app and supported by non-specialist counsellors. The intervention was aimed at a target population of secondary school students in India during the COVID-19 pandemic. POD Adventures (PRIDE, Sangath, India, www.podadventures.in) is part of the PRIDE research programme (2016–2022), which was designed to address the scarcity of evidence-based interventions for common adolescent mental health problems in India, and low-resource settings more broadly. PRIDE involved the development and evaluation of a suite of transdiagnostic psychological interventions to be delivered by non-specialist (‘lay’) counsellors in under-resourced school settings.^20–22^

POD Adventures is grounded in stress-coping theory,^23^ with a mechanistic focus on problem-solving. It was conceptualised as an open-access, early intervention to promote adaptive coping for psychosocial problems, and thus mitigate risks for developing more severe and socially disabling mental health problems in the longer term. The app was designed with adolescents, using a person-centred approach.^21^ The intervention integrates brief guidance from a lay counsellor with self-guided digital content from an app, in line with findings that human facilitation can enhance engagement with, and outcomes of, digital mental health interventions.^16,24,25^ Co-design workshops with young people and iterative piloting suggested that the optimal delivery mode for POD Adventures was small group sessions with up to six students working independently on smartphones under the supervision of a counsellor.^21^ This offline, school-based format was evaluated in 2019–2020 as part of an uncontrolled cohort study (*N* = 248). Findings showed that the intervention was highly acceptable, engaging and feasible to deliver in school settings. Indicative clinical outcomes showed significant reduction in problem severity and mental health symptoms after 4 and 12 weeks.^26^ The timing of the COVID-19 outbreak meant that a planned randomised controlled trial designed to evaluate this offline, in-school mode of delivery of POD Adventures had to be modified to fit around extended school closures, which were instigated in India from March 2020 onward (and remained in place for nearly 2 years). The specific objectives of this modified trial were to assess whether the feasibility and acceptability of POD Adventures would be replicated when delivered online and with remote telephone-based support.

## Method

### Trial design

We conducted a parallel, two-arm, individually randomised controlled pilot trial with outcomes assessed at two timepoints: baseline and 6 weeks post-randomisation. Originally designed as a full-scale trial intended to take place in person from June 2020, we modified the original protocol into a remotely delivered online pilot trial before trial registration or any participants enrolling in the original trial.

### Ethics

The authors assert that all procedures contributing to this work comply with the ethical standards of the relevant national and institutional committees on human experimentation and with the Helsinki Declaration of 1975, as revised in 2008. All procedures involving human patients were approved by the Institutional Review Boards of Sangath (the implementing organisation in India) (approval number PG_2020_69), Harvard Medical School (the sponsor), London School of Hygiene and Tropical Medicine (collaborator) and the University of Sussex (collaborator). This trial was registered at ClinicalTrials.gov (identifier NCT04672486). Additional permissions were obtained from all participating schools. The pilot protocol has been previously published.^27^ Trial findings have been reported according to CONSERVE (CONSORT and SPIRIT Extension for RCTs Revised in Extenuating Circumstance) guidelines for trials, modified for COVID-19.^28^

#### Key modifications

Table 1 summarises key protocol modifications to accommodate online delivery. These modifications were agreed in consultation with the Trial Steering Committee.

**Table 1:** Modifications for online delivery

Protocol item	Original protocol	Modifications owing to COVID-19
1. Methods of recruitment	– Sensitisation sessions conducted for school staff and students in person – The process of study enrolment was in person via the researcher in the school	– Sensitisation sessions conducted for school staff and students, either in person or remotely via virtual classroom – The process of study enrolment was online
2. Intervention delivery	– The POD Adventures app was offered on researcher-provided smartphones on school premises during designated times – Intervention on-boarding (at intervention start) and review (at completion of the intervention) conducted either individually or in small groups – On-hand troubleshooting help for technical or content issues available during intervention sessions in school – No usage reminders to students as intervention was provided in school and on researcher's device – No tailored in-app encouragement offered	– The POD Adventures app made available for remote online download on participant's personal device – Intervention on-boarding and review sessions were conducted individually via telephone, with the use of on-boarding and review videos available on the study website – Troubleshooting guides available on the POD website for troubleshooting the app, POD website and game installation – Toll-free number and counsellor number for any other help needed (telephone delivered) – Notifications and reminders for download, weekly usage reminder and reminder for no log in for 5 days programmed – Automated weekly push notifications to offer in-app encouragement and motivation
3. Study design	– Planned as a fully powered, randomised controlled trial with 522 participants, to be conducted on school premises	– Modified to reflect a pilot trial design. Study objectives relate to testing acceptability and feasibility of online delivery of the intervention

### Setting and participants

#### Setting

The trial was conducted during partial (comprising optional attendance in person at school on a 50% basis, as decided by students/parents) and complete COVID-19 school closures between December 2020 and May 2021 in 11 co-educational, government-aided, English-medium secondary schools in Goa, India, with an overall sampling frame of approximately 2500 students. Schools had an average of 230 students within grades 9–12. Goa is one of India's most urbanised states, and offered a suitable context in which to evaluate an online intervention intended for low-resource settings. Goa was also the setting of an earlier uncontrolled evaluation of the offline version of POD Adventures.^26^ The schools comprised adolescents from both centrally located urban and remote rural areas of the state.

#### Participants

We recruited participants who (a) were enrolled in grades 9–12 (ages 13–19 years) in collaborating schools; (b) had access to an internet-enabled Android smartphone with a valid telephone number for the duration of the pilot trial; (c) were able to read and understand English as a primary language and (d) were willing to provide written assent/consent (including from a parent/guardian (‘caregiver’) for participants aged <18 years). We excluded students who were unable to comprehend the intervention materials (e.g. owing to a reading or hearing disability or inability to comprehend English) or were identified as having an elevated risk of self-harm or suicide and requiring external referral, based on a brief screening questionnaire and follow-up structured interview during study enrolment.

#### Recruitment

Recruitment involved a brief 20- to 30-min sensitisation session delivered to individual classes either online (via virtual classrooms) or, where social distancing policies allowed, in school with a slideshow and brief video containing information about the study; and, where feasible, distribution of a downloadable or printed information flyer via school-moderated email/WhatsApp groups to enrolled students, explaining the study and how to participate.

#### Enrolment

Interested students were directed to visit the study website from their homes and using their own or borrowed devices. The website could be accessed in a language of their choice (English, Hindi or Konkani). Students first completed a short eligibility assessment consisting of questions related to their age, class and language. Eligible students were then prompted to watch an animated video and/or read information about what study participation entailed. Ineligible students were provided with a downloadable information flyer containing details about local and national services and helplines.

Potential participants were guided through a brief online registration process and asked to provide basic demographic details and a telephone number, and to create a password for their use of the study website. This information was sought to be able to contact potential participants to obtain assent or caregiver/parental consent. Following registration, assent/consent was obtained through a web-based consent form via the study website, which was e-signed and dated by participants and the caregiver/parent. Consent was obtained from participants aged ≥18 years and assent from those aged <18 years. For participants aged <18 years, web-based caregiver/parental consent was followed by a confirmatory telephone call from the study team within 2 working days. A toll-free telephone helpline was also made available for prospective participants to ask questions or seek technical support for registration.

#### COVID-19 precautions

The research team implemented the study in line with local and national public health guidance and made every effort to minimise in-person visits to schools unless specifically requested by the school authorities. Fieldwork safety training was provided to all study team members.

### Interventions

#### Intervention arm

The POD Adventures intervention comprised an app and brief counsellor guidance via telephone. Participants also received information about local mental health service providers and government provided/affiliated helplines (i.e. enhanced usual care as offered in the control arm; see below).

The content of the POD Adventures app comprises two sections: ‘Adventures’ teaches problem-solving concepts and methods through contextually appropriate stories and games; and ‘My POD’ guides a individual through the application of step-by-step problem-solving procedures for their own prioritised problem(s). The description of the app has been published elsewhere.^21^ The app was offered in English text with English, Konkani or Hindi voiceover options. Formative work conducted in Goa as part of the intervention design process indicated an overall student preference for English as the primary language of the POD Adventures app. Voiceovers in Hindi and Konkani were added to further assist with comprehension for students who spoke one of these local languages at home.^21^ Guidance was offered in a language of the participants’ choice.

The intervention was initiated by watching a 1 min pre-recorded orientation video via the study website and a brief 10–15 min on-boarding telephone call with a counsellor, in which the counsellor offered an overview of the intervention and worked with the participant to identify and prioritise a target problem(s). The counsellor also provided the participant with app download instructions, and participants were expected to download the app from the study website. Participants were then encouraged to work at their own pace through the Adventures content, and apply the steps of problem-solving to their own prioritised problem(s) in the My POD section of the app. During the fourth week of the intervention or after completing both sections of the app, whichever was first, a brief review call was scheduled between the counsellor and participant via text message or a telephone call. The purpose was to discuss the participant's progress, overall learning and their plan for managing future problems.

For the duration of the study, each participant received a weekly reminder via text message containing encouragements to use the app. They also received a notification reminder to use the app if they did not log in for 5 consecutive days. In addition, counsellors proactively made telephone calls to participants who did not use the app despite reminders. On-demand telephone support from a counsellor was offered for addressing technical problems and clarifying app content throughout the study. A troubleshooting guide about installing the app, resetting passwords and online connectivity problems was made available for participants on the study website.

Guidance was provided by multilingual non-specialist counsellors. Counsellors had 2 years of experience in delivering a face-to-face (analogue) problem-solving intervention,^29^ and 1 year of experience in facilitating use of the POD Adventures app in school-based group sessions.^26^ Counsellors received a 4-day training built around a printed intervention manual. Training included an orientation to the intervention and its contents, and detailed instructions about how to conduct a classroom sensitisation session, procedures involved in session-by-session guidance, participant safeguarding and arrangements for providing technical app-related support to participants. The counsellors’ supervision consisted of weekly peer group supervision meetings (lasting approximately 1 h), moderated by a psychologist. In each meeting, counsellors discussed progress of individual participants, reviewed fidelity checklists from the telephone sessions and identified areas where troubleshooting or support might be required by participants.

#### Control arm

Control arm participants received enhanced usual care, comprising a digital flyer with information about and contact details for local mental health service providers and government-provided and -affiliated mental health helplines.

### Measures

#### Participant characteristics

We collected descriptive sociodemographic data about the selected school populations and adolescents registering for the study. Enrolled participants were also asked to respond to four questions about their mobile phone and internet ownership and use.

#### Feasibility

Feasibility of research procedures was assessed with routinely logged frequencies and proportions of eligible/ineligible self-referrals (with reasons for ineligibility), assenting/consenting participants (with reasons for not assenting/consenting), randomised participants (with reasons for not randomising) and completed outcome assessments (with reasons for non-completion).

Feasibility of intervention delivery was assessed with routinely logged frequencies and proportions of participants who logged into the app at least once, completed individual sections of the app and completed the intervention overall (i.e. attended both the on-boarding and review telephone calls and completed both app sections). Granular data on participants’ use of the app was also recorded via integrated analytics software. Exploratory variables of interest included knowledge of problem-solving assessed through multiple-choice quizzes, and self-reported use of problem-solving in real-world situations (extracted from the My-POD section of the app).

#### Acceptability

Participant satisfaction data was intended to be collected from participants in the intervention arm at 6 weeks, using an adapted eight-item participant satisfaction measure that had been used in previous PRIDE studies,^29^ with four additional forced-choice items that asked specifically about the experience of using the POD Adventures app. Qualitative interviews were also planned to investigate participants’ experiences of online research procedures in both trial arms, with additional questions planned about the acceptability of the POD Adventures app and counsellors’ input for intervention arm participants. Interviews were to be conducted via telephone within 2 weeks of completing the follow-up assessment with a subsample of participants, purposively selected from both trial arms.

#### Clinical outcomes

Indicative clinical outcomes were assessed with two validated self-report questionnaires that measure psychosocial problem severity (Youth Top Problems)^30^ and self-reported depression and anxiety (Revised Child Anxiety and Depression Scale – Short Version (RCADS-25)).^31^ Assessments were carried out at two timepoints: baseline (pre-randomisation) and post-intervention follow-up (6 weeks after randomisation).

### Sample size

We used a confidence interval approach for the calculation of sample sizes for external pilot randomised controlled trials,^32^ which recommended a sample size of 70 participants (35 per arm) to estimate the s.d. for a continuous outcome with adequate precision for a pilot RCT.

### Randomisation and blinding

Each participant was allocated a unique, anonymised identification number after registering on the study website. Upon completion of consent, a notification was sent to the study data manager via a secure web portal designed for the study data collection.

The randomisation algorithm was computer-generated and stratified by school grade, using randomly sized blocks of 4, 6 and 8. Randomisation was performed by the data manager on this platform, and the outcome of allocation was communicated to the participants through a telephone call from a researcher and an SMS text message alert, both of which informed the participant to log in to the study website for information about their allocation. The study website consisted of a personalised dashboard that directed the participant to their next step.

Participants and counsellors were not blinded to allocation status. However, other members of the research team (the principal investigator, trial statistician and researchers) remained blind to participation allocation status.

### Data collection

The first participant was enrolled on 28 January 2021, and their 6-week assessment was completed on 4 April 2021. Baseline data was collected via the study website. Participants received an automated SMS alert to initiate the baseline assessment after completing the above-mentioned assent/consent procedures. A researcher additionally contacted participants by telephone to remind them to complete the baseline assessment if it had not been completed within 2 days.

The follow-up assessment was initiated by SMS text message invitation exactly 6 weeks after randomisation. This message was followed up by a telephone call from a researcher following a standardised script that asked participants to complete the assessment. Automated SMS text message reminders were sent to participants every 3 days over the next 2 weeks or until the follow-up assessment was completed on the study website. Researchers made a minimum of four telephone attempts following the due date, with a maximum allowance of 2 weeks.

The research study team received training on conducting remote and in-person recruitment activities, such as sensitisation sessions; participant consent procedures via the study website and telephone; providing telephone reminders for assessments and technical troubleshooting via the toll-free study helpline. Supervision of the research team consisted of weekly meetings (lasting approximately 1 h), moderated by the study coordinator. In each meeting, the team reviewed participant enrolment progress, data collection procedures and any technical difficulties encountered that required troubleshooting.

#### Data security and management

The study was hosted on the servers of Sangath, the implementing organisation based in Goa, India. These servers were encrypted, with data back-ups occurring daily. The study web portal and its associated data were accessible only to authorised and approved personnel.

### Statistical analysis

The statistical analysis was mainly descriptive in nature, aiming to provide estimates of key feasibility and acceptability parameters and indicative clinical outcomes. The outcome measures were summarised at baseline and at 6-week follow-up, by trial arm. These were summarised by mean (s.d.), median (interquartile range), or number (percentage) values overall, and stratified by age, gender and baseline outcome score.

## Results

### Feasibility of research procedures

Overall, 79 sensitisation sessions (12 online, 67 in person) were conducted, reaching a total of 1575 students. From the sensitised sample, 92 referrals (5.8%) were received, all self-initiated by students. Most referrals (*n* = 69, 75%) originated from in-person sensitisation sessions, followed by referral forms via drop boxes placed in schools (*n* = 18, 19.5%). Only five referrals (5.4%) were made through the toll-free helpline following online sensitisation.

From the referred sample, 38 students (41.3%) completed the online eligibility self-screener. One student was excluded because of literacy difficulties, and three were excluded because of lack of access to a smartphone (*n* = 3). No students were excluded because of risk. Of the 34 eligible referrals, 16 (45%) completed consent procedures and the remaining 18 referrals did not enrol. Reasons for non-participation included students who were uncontactable (*n* = 9), unable to access a telephone/internet (*n* = 4), examinations (*n* = 2), problem resolved (*n* = 2) and parent consent denied (*n* = 1). The mean time taken from referral to randomisation was 6.8 days (s.d. = 8.8).

**Fig. 1:**
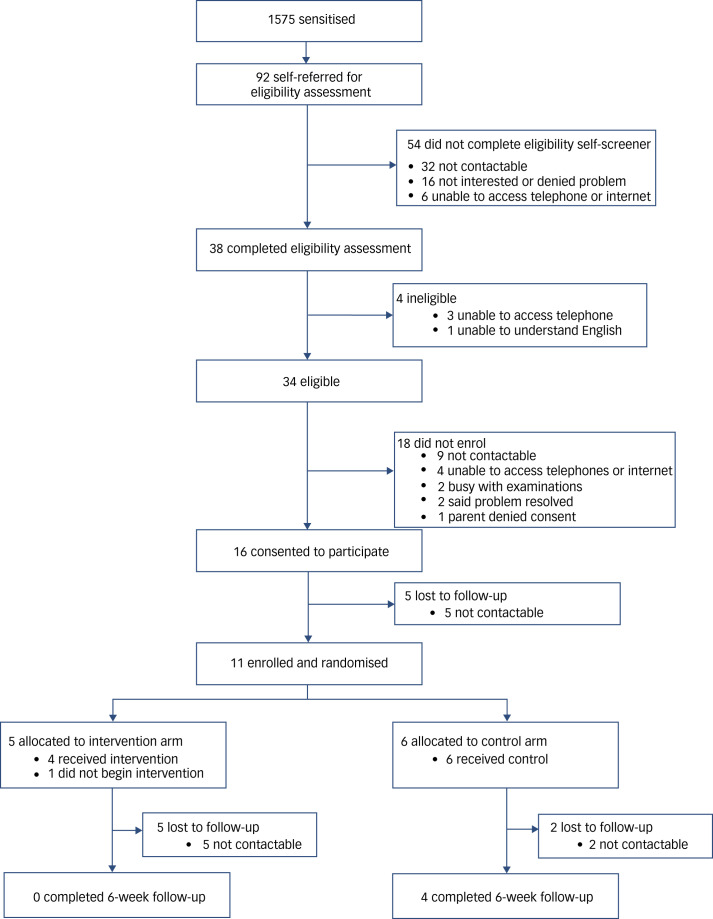
Participant flow diagram.

Overall, 11 participants completed the baseline assessment and were randomised. Five participants were allocated to the intervention arm and six participants were allocated to the control arm (Fig. 1).

### Participant characteristics

Randomised participants were aged 13–19 years (mean 15.3 years, s.d. = 1.0; four male and seven female) (Table 2). All participants had RCADS scores in the normal range (RCADS *T*-score mean for anxiety and depression was 50.7, s.d. = 7.8) (Table 3). Baseline demographic and clinical characteristics were similar across the two study groups. As part of the baseline assessment, participants also completed a brief technology exposure survey. Seven participants (63.3%) reported owning a personal mobile phone and four participants (36.4%) reported having access to a family/shared mobile phone. Ten participants (90.9%) reported using a smartphone and the internet nearly every day and one participant reported use every 3–4 days.

**Table 2:** Participant characteristics

	Intervention arm (*n* = 5)	Control arm (*n* = 6)	Total (*n* = 11)
Gender			
Female	3 (60.0%)	4 (66.7%)	6 (54.5%)
Male	2 (40.0%)	2 (33.3%)	5 (45.5%)
Mean age (years), s.d.	15.0 (0.5)	15.5 (1.3)	15.3 (1.0)
Grade			
9	4 (80.0%)	3 (50.0%)	7 (63.6%)
10	1 (20.0%)	2 (33.3%)	3 (27.3%)
11	0 (0.0%)	0 (0.0%)	0 (0.0%)
12	0 (0.0%)	1 (16.7%)	1 (9.1%)
Referral source			
At classroom sensitisation session	5 (40.0%)	6 (60.0%)	11 (100%)

**Table 3:** Participant baseline characteristics

	Baseline profile (*n* = 11), mean (s.d.)	Intervention arm (*n* = 5), mean (s.d.)	Control arm (*n* = 6), mean (s.d.)
YTP total score	6.5 (1.7)	6.6 (2.1)	6.5 (1.4)
RCADS *T*-score			
Total depression	49.3 (7.6)	46.8 (3.8)	51.3 (9.6)
Total anxiety	52.0 (8.9)	50.6 (4.2)	53.2 (11.9)
Total anxiety and depression	50.7 (7.8)	48.6 (2.9)	52.5 (10.4)

YTP, Youth Top Problems; RCADS, Revised Child Anxiety and Depression Scale – Short Version.

### Feasibility and acceptability of intervention delivery

Process indicators for the intervention group are summarised in Table 4. Two participants did not begin the intervention (i.e. did not attend the on-boarding call with the counsellor or use the app), and no participants completed the intervention. Only one participant completed the first section of the app. Reasons for non-completion were unspecified as participants could not be reached by the study team. For the same reason, no qualitative interviews or participant satisfaction measures were completed.

**Table 4:** Intervention use indicators

Intervention indicators	*N* = 5
Intervention orientation video watched on study website	4 (80%)
On-boarding session with counsellor completed via telephone	3 (60%)
First section of POD Adventures app completed	1 (20%)
Second section of POD Adventures app completed	0 (0%)
Third section of POD Adventures app completed	0 (0%)
Fourth section of POD Adventures app completed	0 (0%)

Data about participants’ use of the app captured via an integrated analytics software was excluded from this analysis because of very low completion of the app and non-completion of sections such as multiple-choice quizzes, or self-reported use of problem-solving in real-world situations.

### Indicative clinical outcomes

No outcome data were available for intervention arm participants, none of whom could be reached to complete assessments.

## Discussion

This study aimed to evaluate the feasibility and acceptability of POD Adventures, an app-based adolescent mental health intervention, when delivered online accompanied by telephone support from counsellors during the COVID-19 pandemic in India. Despite a range of modifications to address barriers arising from COVID-19 restrictions, online remote delivery was not found to be acceptable or feasible for the target population in the study context.

The current results contrast remarkably with feasibility and acceptability findings from a large school-based cohort study (*N* = 248) of POD Adventures, which was delivered offline on school premises before the COVID-19 pandemic. The latter study had comparatively higher rates of self-referral (18.2 *v*. 5.6% in the current study) and intervention completion (93% *v*. no completers in this study). Qualitative interviews with participants further showed that the app was easy to use, engaging and helpful in solving their problems when used offline, and the brief guidance provided by counsellors was experienced as adequate and helpful whether provided individually or in small groups.^26^

In the current study, we additionally found that a very small proportion of self-referred students ultimately completed assent/consent procedures, and no participants in the intervention arm completed the intervention or outcome assessments despite the motivators for the intervention and the efforts by the research team. The absence of qualitative data about barriers to study participation and intervention use make it difficult to identify the extent to which participation challenges resulted from difficulties with smartphone or internet access, specific concerns about accessing a mental health intervention online (e.g. related to privacy or helpfulness) and/or the timing of the study. Regarding study timing, it is notable that the research took place during the especially severe second COVID-19 wave in India, which included widespread lockdowns and school closures in the context of overwhelmed health systems.^1,6^ Potential participants may have been concerned about their own physical health or illness affecting family members.

The findings of this study are broadly aligned with research related to the non-adoption or scale-up of many promising technological innovations in healthcare.^33^ Greenhalgh et al, in three reviews of technology diffusion and implementation,^33–35^ have shown that the implementation of a new technology as part of changes to healthcare services is inherently very challenging. These reviews highlight that those innovations requiring changes in organisations or the wider care system have a poor track record of adoption because of the dual challenge of non-adoption by individuals and difficulties with spread or scale-up. They further emphasise that it is not only individual factors that make or break a technology implementation effort but the dynamic interplay between these factors. The more complex an innovation or the setting in which it is introduced, the less likely it is to be successfully adopted or scaled up. They also emphasise that methodologically robust randomised controlled trials alone will not elucidate these complex interactions, and emphasise the need for more studies that are interdisciplinary, non-deterministic, locally situated and designed to examine the recursive relationship between human action and the wider system context.^36^ Further, findings from the current study are consistent with those from recent reviews that show that digital interventions may achieve greater uptake and sustained engagement when delivered in structured and guided settings, such as schools or clinics.^16,18,19^ Furthermore, these reviews have concluded that interventions involving educational programmes completed in the participant's own time (i.e. at home without supervision) are not effective. Li et al found that prior experiences of accessing mental health counselling may make students more open to online services,^37^ a finding that was not relevant to the present sample because they had limited experiences of in-person mental health services. Given the prior findings that showed high uptake, adherence and engagement with POD Adventures when delivered in schools,^26^ it seems likely that an online study format and remote delivery, rather than the app content, were the biggest barriers to study participation and engagement. Further, a systematic review by Garrido et al found that study drop-out rates for digital interventions for depression and anxiety with young people tended to relate more to recruitment methods, especially in the case studies completed by participants at home/in their own time, than to non-engagement of the interventions themselves.^16^

Findings from the implementation of remote learning approaches in schools in LMICs around the world have revealed extremely low rates of smartphone and internet access, ranging from 2 to 6% of young people with access at home. This has impeded learning and school participation throughout the COVID-19 pandemic.^38^ In the current study, self-referring students who were unable to enrol in the study reported lack of access to a smartphone or internet as the most common reason for non-participation. Even among those participants who did enrol in the study, it is possible that they faced recurring or intermittent difficulties with accessing a smartphone and/or internet connectivity. For intervention arm participants in particular, telephone guidance and text messages may not have been readily accessible because of sporadic telephone access.

Another key barrier may have been limited sensitisation in a sample with little to no prior experiences of formal mental health interventions. It is possible that the brief classroom sensitisation session provided as part of recruitment was insufficient in building an understanding of the intervention features or potential benefits when delivered online.

Recent research shows that enhancing mental health literacy and addressing concerns about the implications of making use of online help-seeking (such as concerns about privacy, that it may be too impersonal or that the help would be unreliable and untrustworthy) may help build demand from young people.^37,39,40^

### Implications for future research

This study suggests there may be a gap between the potential that online digital interventions offer and the reach and uptake of these technologies, especially for adolescents from LMICs,^41^ emphasising the ongoing need for in-person interventions as well as the development and evaluation of technologies that are context-specific; for example, use of web browser-based rather than app-based interventions, as they do not require downloading or regular updates or work on any smartphone device.^14,41^

There is also a pressing need to distinguish digital mental health interventions by their content and delivery characteristics. Findings from this pilot trial suggest that online delivery of POD Adventures was not feasible, despite earlier evidence that the content of the app was useful, appropriate and potentially effective in the same population. Furthermore, distinct adaptations that account for population-specific needs of adolescents (e.g. technology ability and access, literacy levels, media and language preferences) are needed.^41^ Future evaluations of POD Adventures (and mental health apps more generally) should systematically record and report use of different languages where these can be selected by users. Interventions which explicitly aim to reduce demographic disparities related to intervention retention (e.g. the use of very brief digital interventions such as single session rather than multiple sessions) should be evaluated.^42,43^ Finally, judicious use of technology for trial procedures and the use of hybrid approaches, which include in-person interaction at school, may help to conduct evaluations.^44^

In conclusion, findings from this pilot trial are inconclusive about whether the key barriers to adolescent participation were a result of difficulties accessing online research procedures, intervention delivery or a combination of both, which may have been exacerbated by COVID-19 pandemic conditions. More studies with this age group and in similar settings are needed to establish the generalisability of these findings.

## Data Availability

The data that support the findings of this study are available from the corresponding author, P.P.G., upon reasonable request.
